# Three-Dimensional Imaging Method for Array ISAR Based on Sparse Bayesian Inference

**DOI:** 10.3390/s18103563

**Published:** 2018-10-20

**Authors:** Zekun Jiao, Chibiao Ding, Longyong Chen, Fubo Zhang

**Affiliations:** 1 National Key Laboratory of Science and Technology on Microwave Imaging, Beijing 100190, China; ustcjzk@gmail.com (Z.J.); lychen@mail.ie.ac.cn (L.C.); zhangfubo8866@126.com (F.Z.); 2 Institute of Electronics, Chinese Academy of Sciences, Beijing 100190, China; 3 University of the Chinese Academy of Sciences, Beijing 100049, China

**Keywords:** three-dimensional imaging, array ISAR, synthesis scatterers, elastic net regression, sparse Bayesian inference

## Abstract

The problem of synthesis scatterers in inverse synthetic aperture radar (ISAR) make it difficult to realize high-resolution three-dimensional (3D) imaging. Radar array provides an available solution to this problem, but the resolution is restricted by limited aperture size and number of antennas, leading to deterioration of the 3D imaging performance. To solve these problems, we propose a novel 3D imaging method with an array ISAR system based on sparse Bayesian inference. First, the 3D imaging model using a sparse linear array is introduced. Then the elastic net estimation and Bayesian information criterion are introduced to fulfill model order selection automatically. Finally, the sparse Bayesian inference is adopted to realize super-resolution imaging and to get the 3D image of target of interest. The proposed method is used to process real radar data of a Ku band array ISAR system. The results show that the proposed method can effectively solve the problem of synthesis scatterers and realize super-resolution 3D imaging, which verify the practicality of our proposed method.

## 1. Introduction

With further investigation of space resources in recent years, the surveillance of space target has gained much more attention among institutes around the world. Compared with the traditional two-dimensional (2D) inverse synthetic aperture radar (ISAR) images, three-dimensional (3D) radar images can provide energy distribution of scatterers and more details of 3D geometric features, which will finally contribute to target recognition [1]. Toward this end, various method are proposed to reconstruct 3D images of targets [2,3,4]. These methods can be divided into two categories.

Combined with the interferometric technique, the first category is the interferometric ISAR (InISAR) technique [5,6]. In the framework of InISAR, the elevation information was obtained by comparing the phase difference of the received signals from two antennas. To extract 3D information from 2D ISAR images by interferometric technique, various methods are proposed [6,7,8,9,10,11]. In [7], 3-D ISAR imaging method with a sparse array is proposed and the CLEAN technique is adopted to extract strong artificial scatterers caused by high sidelobes. In [8,9], three-dimensional imaging method using a two-dimensional antenna array is proposed. In [10,11,12], compressed sensing based approaches for 3D ISAR imaging are proposed. In [13], polarimetric inverse synthetic aperture radar (ISAR) for classification of small boats is proposed. In [14,15], a fully polarimetric three-dimensional imaging method is proposed and the 3D visualization methods are also presented. In the literature, ISAR image pairs are collected at slightly different elevation angles and interferometrically processed into 3D images. Finally the 3D reconstruction results are overlaid on to a CAD model of the targets. In [16], three-dimensional interferometric ISAR imaging method is proposed and the problem of scattering center extraction and the 3D reconstruction alignment are discussed. However, there are mainly two shortcomings. The first is that the coordinate-registration problem mentioned in [9]. The second is the problem of synthesis scatterers. Since the ISAR image is a projection of the 3D target onto the imaging projection plane, scatterers with the same range and Doppler frequency will be projected in the same pixel and hard to distinguish. These scatterers are known as the synthesis scatterers, which is also called angular glint effect [8]. Traditional InISAR technique does not have the ability to distinguish multiple dominant scatterers from a synthesis scatterer. The reason for this phenomenon is that the resolution in the elevation direction depends on the baseline length. In order to get a finer image, the antenna array can be very large which make it difficult for practical applications.

The second class of 3D reconstruction is based on the sequence ISAR approach [17,18,19,20,21,22]. In [17], three-dimensional model extraction with sequence ISAR images is proposed with the assumption that scatterers can be correctly registered from frame to frame. In [18], 3D scatterer models are extracted from a sequence of ISAR images and used for ship classification. In [19], the radar system first obtains a sequence of 2D ISAR images. Then feature tracking methods such as the Kanade-Lucas-Tomasi (KLT) algorithm are firstly adopted to extract the feature points from a sequence of ISAR images [23]. Then the feature points from different images are matched and the 3D positions of these scatterers can be retrieved by orthographic factorization method (OFM) [24]. Joint cross-range scaling algorithm via sequential ISAR images can be found in [25]. However, this kind of method needs a long observation time and the incoherency caused by the long imaging time will affect the 3D reconstruction result [16]. Besides, for both of the two categories, most of the literatures so far focus on simulation and signal processing of simple target with only a few point scattering centers. However, in real situation an aircraft is always composed of thousands of scatterers which would need a lot of work on imaging processing.

To solve the problems aforementioned, varies approaches are proposed. In [2], three-dimensional reconstruction method using two perpendicular antenna arrays to measure the positions of scatterers of a synthesis scatterer is proposed. A similar approach is proposed in [9] that a multiple-input multiple-output (MIMO) is used and the elevation position is estimated by spectral analysis. However, the resolution in the elevation direction is still restricted by the limited baseline length under this framework. With the development of compressed sensing (CS), the super-resolution ability of sparse recovery methods shows its advantage over traditional spectral analysis algorithms. In [26,27], CS based super-resolution reconstruction methods are proposed in SAR tomography and an elevation resolution better than the Rayleigh limit can be achieved. In [11], CS technique is adopted in ISAR imaging. Inspired by the result in SAR tomography, we proposed a 3D imaging method to overcome the problems of synthesis scatterers and realize super-resolution 3D reconstruction in this paper. Since the dominant scatterers in a single range-Doppler unit are usually sparse in the elevation direction, the compressed sensing (CS) technique is utilized here. The sparse assumption here is based on the results in [11,26] and the 3D reconstruction result with measured radar data prove its validity. Under this framework, multi-channel ISAR images are firstly obtained by using a sparse linear array, which will reduce the imaging duration compared with the sequence ISAR technique. After image registration and phase compensation, the elastic net estimation [28] along with Bayesian information criterion (BIC) [29] is applied to estimate the number of dominant scatterers without any prior information. With this estimation as a prior, the sparse Bayesian inference method [30] is then used to obtain an accurate estimation of the reflectivity function along the elevation direction. After these manipulations, the 3D model of targets can be reconstructed. Simulation results prove the superiority of the proposed algorithm over other existing three-dimensional recovery algorithms such as SVD-Wiener and “Scale-down by L1 norm Minimization, Model selection, and Estimation Reconstruction” (SL1MMER). In addition, Bayesian Cramér-Rao bound (BCRB) and the Mean Square Error (MSE) are compared to verify effectiveness of the proposed method. Besides, measured radar data are used to extract 3D model of the airplane, which validates the effectiveness and feasibility of proposed method.

This paper is organized as follows. The 3D imaging model with the array ISAR system is presented in Section 2. In Section 3, an algorithm for 3D imaging based on sparse Bayesian inference is proposed and the Bayesian Cramér-Rao bound for scatterers’ position estimation is also presented. In Section 4, both simulation results and experimental results are presented to analyze the imaging performance. Finally, conclusions are drawn in Section 5.

## 2. 3D Imaging Model of the Array ISAR System

**Figure 1:**
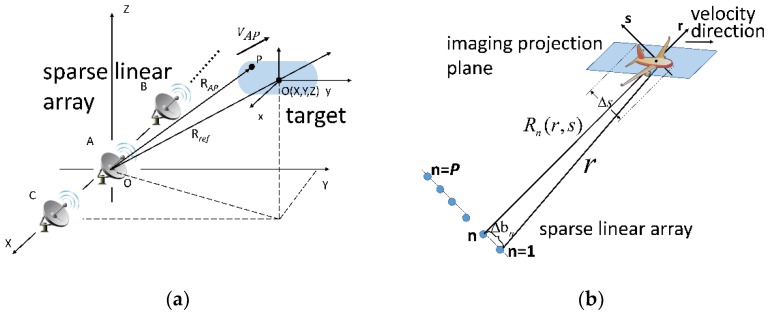
The 3D imaging geometry of array ISAR. (**a**) Overview of the ISAR imaging geometry; (**b**) the imaging model in the range-elevation plane.

The 3D imaging geometry of the array ISAR system is depicted in Figure 1a. As can be seen, the radar array is composed of *M* transmitters and *N* receivers, which means that there are *P* (=*MN*) antenna phase centers (APCs) based on the MIMO technique. The *i*th element is located at (*x_i_*, 0, 0). Here we assume that the target bears a constant velocity in the direction of ***v***. With the *MN* APCs each being an independent channel, we can conduct ISAR imaging using the echo signal of these channels and finally get *P* 2D ISAR images. Taking the ISAR image corresponding to the APC at the origin in Figure 1a as an example, the complex pixel value of scatter P is as follows:

(1) $$S_{P}(f,\;f_{m})=A\sin c\left[T_{p}(f+\frac{2\gamma }{c}R_{\Delta \mathrm{AP}})\right]\sin c\left[T_{1}(f_{m}+\frac{2V_{\mathrm{AP}}}{\lambda })\right]\exp(-j\frac{4\pi }{\lambda }R_{\Delta \mathrm{AP}})$$

in which $R_{\Delta \mathrm{AP}}=R_{\mathrm{AP}}-R_{ref}$ and $V_{\mathrm{AP}}$ is the radial velocity of P with respect to radar A. $T_{p}$ is the pulse width and $T_{1}$ is the overall coherent processing interval. f and $f_{m}$ are the fast-time frequency and Doppler frequency after Fourier transform, respectively. The first two sin*c* functions represent the scatterer’s range and Doppler information. When multiple scatterers have the same range and Doppler frequency, these scatterers will fall into the same pixel and form a synthesis scatterer in the ISAR image. Traditional InISAR technique cannot distinguish these different scatterers from the synthesis scatterer, which leads to the deterioration of 3D imaging performance. Based on the above equation, contribution of the scatterer P to the complex value of a focused range-Doppler pixel is the phase item at the end of Equation (1). Based on these observations, the resolution power in range and Doppler dimension is provided by the ISAR technique while that in the elevation dimension is provided by sparse recovery methods.

As mentioned above, the phase of a pixel of the ISAR image is the last item of Equation (1). Figure 1b presents the imaging model in the elevation dimension, i.e., the dimension perpendicular to the imaging projection plane (IPP). After multi-channel ISAR images registration, for a same range-Doppler unit, the complex value of this unit in the *n*th ISAR image can be expressed as follows according to Equation (1):

(2) $$y_{n}=\int_{s_{\min}}^{s_{\max}}\sigma (s)\exp\left[-j\frac{4\pi }{\lambda }R_{n}(s)\right]ds$$

which is the integral of the reflectivity function along the elevation direction and [*S_min_*, *S_max_*] is the elevation extent. Since there are *P* ISAR images in total, after image registration, we can get *P* measurements for each range-Doppler unit. Consider the antenna at the origin as a reference, the continuous model in Equation (2) can be approximated by discretizing *s* into *L* sampling grids. After adopting Fresnel approximation and multiplying the received signal with the phase factor corresponding to the echo of the elevation center, the discretized signal model is as follows:

(3) y=Φ⋅σ+n

in which ***y*** is a *P* × 1 measurement vector and ***n*** is white Gaussian noise. The element of Φ is $\Phi_{i,j}=exp\left(-j\frac{4\pi }{\lambda }\frac{\Delta b_{i}s_{j}}{r}\right)$ in which *r* is the range, *b_i_* is the position of *i*th APC and *s_j_* is the *j*th sampling point. σ is the reflectivity function in the elevation direction with *s_j_* (*j* = 1, 2, …, *L*) being the sampling points. Hence, this formula can be seen as the spatial Fourier transform of the reflectivity function. To solve the problem of synthesis scatterers and realize 3D imaging, it is equivalent to solve the underdetermined problem of (3). If the reconstructed reflectivity function can be exactly extracted from Equation (3), the dominant scatterers in the same range-Doppler unit can be successfully distinguished and the 3D model of targets can be obtained.

## 3. Array ISAR Three-Dimensional Imaging Method Based on SBI

**Figure 2:**
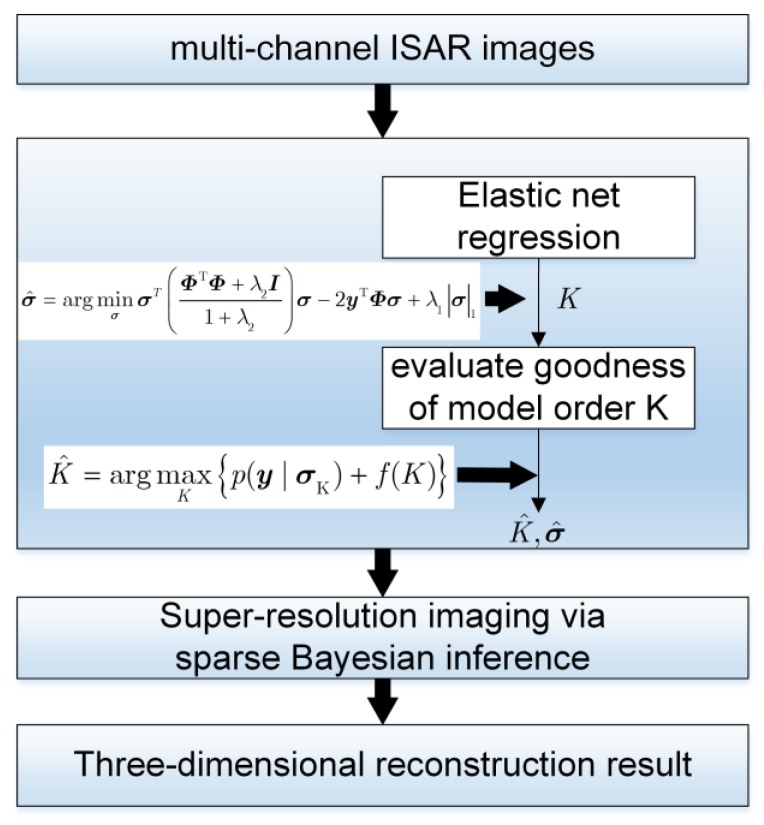
Flowchart of proposed array ISAR 3D imaging method.

Based on the imaging model introduced in Section 2, a sparse Bayesian inference based three-dimensional imaging method for array ISAR system is proposed in this part. It is worth mentioning here why the sparse Bayesian inference is suitable for array ISAR imaging. Inspired by the results in [31], we assume that the number of dominant scatterers in the same range-Doppler pixel is small. According to the three-dimensional imaging model in multi-baseline SAR interferometry, scatterers in the same range-azimuth resolution cell are from the intersections between 3D surface of the target and the equidistant sphere. For this reason, the number of dominant scatterers in layover areas is usually small. Here we refer reader to [31] for more details about the multi-baseline SAR interferometry model. Because the array ISAR 3D imaging is similar to the imaging model in [26], we assume that the dominant scatterers are sparse in the elevation direction and thus the sparse recovery technique can be adopted. The overall procedure of this method is as follows. When echo signal is received, multi-channel ISAR imaging and image registration will be conducted in sequence. After these manipulations, we first adopt elastic net estimation together with Bayesian information criterion (BIC) to realize model selection, i.e., to evaluate the number of dominant scatterers *K* in the same range-Doppler unit. Then we use sparse Bayesian inference technique to fulfill super-resolution imaging in the third dimension. Finally the 3D scattering model of target is extracted after point cloud stitching. The flowchart of our proposed method is depicted in Figure 2.

### 3.1. Model Order Selection Based on Elastic Net Regression

In order to get the distribution of dominant scatterers in the angular glint area, we need to extract σ from Equation (3). Toward this end, the top priority is to estimate the number of dominant scatterers in a range-Doppler unit. Inspired by the idea of SL1MMER algorithm proposed in [18], we first use the elastic net method to get a coarse estimation, then the BIC is used to realize model order selection and find the optimal number *K*.

With limited number of antennas, the number of sampling points *L* in the third dimension is always much larger than the number of APCs *P*, i.e., *L* ≫ *P*. To solve this underdetermined equation, different solutions are proposed with different penalty terms, for example, Lasso estimation with L1-norm penalty and Ridge estimation with L2-norm penalty. To combine advantages of the two approaches mentioned above, Trevor Hastie proposed the elastic net (ELN) regression approach [28]. For the purpose of solving Equation (3), the ELN method uses the following expression:

(4) $$\hat{\sigma }=\arg\;\underset{\sigma }{\min}\;\sigma^{T}\left(\frac{\Phi^{T}\Phi +\lambda_{2}I}{1+\lambda_{2}}\right)\sigma -2y^{T}\Phi \sigma +\lambda_{1}{\left|\sigma \right|}_{1}$$

Compared with solutions by Lasso and Ridge mentioned above, the elastic method accommodates the array ISAR 3D imaging better. On the one hand, since the dominant scatterers are sparsely distributed in the elevation direction, the L1-norm regularization term ensures a sparse solution and provides the SR capability. On the other, there exists multicollinearity among columns of Φ since it is composed of a set of over-complete bases. The ELN method can successfully solve this problem via L2-norm penalty. Denote $\gamma =\lambda_{2}/\left(1+\lambda_{2}\right)$ and $\sum =\Phi^{T}\Phi$, we have:

(5) $$\frac{\Phi^{T}\Phi +\lambda_{2}I}{1+\lambda_{2}}=(1-\gamma )\sum +\gamma I$$

In linear discrimination analysis theory, the accuracy can be improved by using the weighted sum of identity matrix and the autocorrelation matrix of a set of overcomplete bases, as shown in Equation (5). It is worth mentioning that $\lambda_{1}$ and $\lambda_{2}$ need to be adjusted according to noise level and number of measurements in order to obtain a superior performance.

After solving the problem of Equation (4), we can get an initial estimation of the reflectivity distribution in the elevation direction in the area where angular glint effect appears. However, the result always contains some outliers due to the noise. In order to get the 3D reconstruction result with high accuracy, it is necessary to further realize model order selection after obtaining the initial result. Based on max a posterior (MAP) theory and Bayesian information criterion (BIC) [29], we proposed a model order selection scheme. The details are as follows. Assuming that a certain synthesis scatterer is composed of *K* dominant scatterers and the corresponding reflectivity coefficient vector is $\sigma_{K}$, the noise n obeys the complex Gaussian distribution, i.e., n~CN(n|0,ηI). Then the likelihood function can be expressed as:

(6) $$p(y|\sigma_{K})=C\exp(-\frac{\eta {\left\|y-\Phi \sigma_{K}\right\|}_{2}^{2}}{2})$$

in which C is a constant. In order to avoid overfitting, a penalty item f(K) is considered based on the BIC. By this means, the model order *K* can be evaluated through the following expression:

(7) $$\begin{array}{l} \hat{K}=\arg\;\underset{K}{\max}\left\{p(y|\sigma_{K})+f(K)\right\}\\ \;=\arg\;\underset{K}{\min}\left\{\eta {\left\|y-\Phi \sigma_{K}\right\|}_{2}^{2}+\beta K\ln P\right\} \end{array}$$

After finishing the selection of model order *K*, we now get the accurate number of dominant scatterers in a single synthesis scatterer. The results obtained in this step serve as a prior information in the following sparse Bayesian inference steps.

### 3.2. 3D Reconstruction Based on Sparse Bayesian Inference

#### 3.2.1. Elevation Reconstruction Model with Off-Grid Mismatch

Considering the signal model defined in Equation (3), the definition of sensing matrix Φ is based on the discretization of sampling points in the elevation direction. Traditional sparse recovery methods such as OMP and BP are all under the assumption that the true scatterers are exactly located on the predefined sampling grids as mentioned above. However, this assumption will not always hold true in real scene.

Assuming that the sampling points in the elevation direction are $s_{j}\;\left(j=1,\;2,\;\ldots ,\;L\right)$ and that a dominant scatterer’s true position is ${\hat{s}}_{k}\notin \left\{s_{j}\;\left(j=1,\;2,\;\ldots ,\;L\right)\right\}$, the off-grid mismatch emerges. This mismatch will introduce multiplicative noise which is hard to handle by traditional sparse recovery algorithms. In order to get a superior imaging performance, we first establish an optimized sparse recovery model which takes the mismatch problem into consideration.

As mentioned above, ${\hat{s}}_{k}$ is a scatterer’s true position and its corresponding nearest sampling point is $s_{k}^{\prime }\in \left\{s_{j}\;\left(j=1,\;2,\;\ldots ,\;L\right)\right\}$, then ${\hat{s}}_{k}=s_{k}^{\prime }+\delta_{s_{k}}$ in which $\delta_{s_{k}}$ is the off-grid mismatch. Rename the original sensing matrix Φ in Equation (3) as $\Phi_{0}=\left[\varphi \left(s_{1}\right),\;\varphi \left(s_{2}\right),\;\ldots ,\;\varphi \left(s_{L}\right)\right]$ in which $\varphi \left(s_{k}\right)$ is the steering vector of sampling point $s_{k}$, we make a linear approximation of the scatterer’s true steering vector $\varphi \left({\hat{s}}_{k}\right)$ using the first order Taylor series expansion as:

(8) $$\varphi \left({\hat{s}}_{k}\right)\approx \varphi \left(s_{k}^{\prime }\right)+\varphi^{\prime }\left(s_{k}^{\prime }\right)\left({\hat{s}}_{k}-s_{k}^{\prime }\right)$$

in which $\varphi^{\prime }\left(s_{k}^{\prime }\right)$ is the derivative of $\varphi \left(s_{k}^{\prime }\right)$ with respect to $s_{k}^{\prime }$. Thus we can get the approximated off-grid signal model:

(9) $$y=(\Phi_{0}+\Phi_{1}\cdot \Delta s)\sigma +n$$

in which $\Delta s=\mathrm{diag}\left(\delta_{s}\right)$, $\delta_{s}=\left[\delta_{s_{1}},\;\delta_{s_{2}},\;\ldots ,\;\delta_{s_{L}}\right]$ and $\Phi_{1}=\left[\varphi^{\prime }\left(s_{1}\right),\;\varphi^{\prime }\left(s_{2}\right),\;\ldots ,\;\varphi^{\prime }\left(s_{L}\right)\right]$. The signal model in Equation (9) takes the mismatch between scatterer’s true position and the sampling grids into consideration and will accommodate the real scene better. In the following section, a sparse Bayesian inference based method is introduced to extract Δs and σ from Equation (9).

#### 3.2.2. 3D Reconstruction Algorithm Based on SBI

To solve the sparse recovery problem in Equation (9), we adopt the sparse Bayesian inference method [30,32] to overcome the shortcomings mentioned above. With the estimated number of dominant scatterers *K* and an initial estimation $\sigma_{K}$ obtained in Section 3.1 as prior information, we adopt this algorithm to fulfill sparse recovery in the elevation direction. In the following we will briefly explain how this method is used in our 3D imaging process.

Under the framework of sparse Bayesian inference, the estimation of Δs and σ can be solved through MAP estimation. According to previous literatures, it is reasonable to assume that scatterers’ reflectivity coefficients obey complex normal distribution, i.e., σ~CN(σ|0,Λ) where Λ=diag(α). In order to utilize the property of conjugate priors [30], here we assume that the hyper-parameter α obeys Gamma distribution $p\left(\alpha ;\rho \right)=\prod \Gamma (\alpha_{i}|1,\rho )$. The white Gaussian noise ***n*** is complex Gaussian, i.e., n~CN(n|0,ηI) in which η is the noise variance. Same as before, we assume that (η;c,d)= Γ(η|c,d). Since the scatterers are distributed continuously in real scene and there is no prior information about the off-grid mismatch $\delta_{s}$, we assume that $\delta_{s}$ obeys the uniform distribution:

(10) $$\delta_{s}\sim U([-\rho_{s}/2,\rho_{s}{/2]}^{L})$$

in which $\rho_{s}$ is the sampling interval. According to the Bayesian formula, the posterior probability density function can be obtained:

(11) $$\begin{array}{l} p(\sigma ,\Delta s,\eta ,\alpha |y)=p(\sigma ,\Delta s,\eta ,\alpha ,y)/p(y)\\ p(y)=∭p(\sigma ,\Delta s,\eta ,\alpha ,y)d\sigma d\Delta sd\alpha d\eta \end{array}$$

With the posterior probability density function, we can obtain the estimation of reflectivity coefficient vector σ, the off-grid mismatch $\delta_{s}$ and the noise variance η based on the MAP estimation.

According to Equation (11), we need to get the explicit expression of p(y) while the integral is hard to calculate. So we use the variational inference technique to get an approximated analytic expressions. The results are presented here and we refer readers to [32] for more details about the variational inference technique. The estimation of σ, Δs, η, α can be calculated according to the following expressions:

(12) $$\begin{array}{l} \sigma =\eta \Sigma \Phi^{H}y;\\ \alpha_{i}=\frac{\sqrt{1+4\rho E\left\{{\left\|\sigma_{i}\right\|}_{2}^{2}\right\}}-1}{2\rho }(i=1,2\ldots L);\\ \eta =\frac{E\left\{{\left\|y-\Phi \sigma \right\|}_{2}^{2}\right\}+d}{P+c-1} \end{array}$$

in which $\Sigma ={\left[\eta \Phi^{H}\Phi +\mathrm{diag}(1/\alpha )\right]}^{-1},\;\Phi =\Phi_{0}+\Phi_{1}\cdot \Delta s$. As for the off-grid mismatch $\delta_{s}$, it can be calculated by solving the following convex optimization problem:

(13) $$\delta_{s}=\arg\min_{\delta_{s}}\{\delta_{s}{}^{T}B\delta_{s}-2x^{T}\delta_{s}\}$$

with:

(14) $$\begin{array}{l} B=ℜ(\bar{\Phi_{1}{}^{H}\Phi_{1}}⊙(\sigma \sigma^{H}+\Sigma ))\\ x=ℜ\{diag(\bar{\sigma })\Phi_{1}{}^{H}(y-\Phi_{0}\sigma )-diag(\Phi_{1}{}^{H}\Phi_{1}\Sigma )\} \end{array}$$

Equations (11)–(14) together suggest an iterative approach for estimating the unknowns. The iteration ends when the number of iteration exceeds a threshold or the relative change of two successive iteration is less than a predefined constant ε, i.e., $\frac{∥\sigma^{\left(i+1\right)}-\sigma^{\left(i\right)}{∥}_{2}^{2}}{∥\sigma^{\left(i\right)}{∥}_{2}^{2}}<\varepsilon$. With the results obtained in Section 3.1 as initial input, we can fulfill super-resolution 3D reconstruction through iteration which is presented in this section.

#### 3.2.3. Bayesian Cramér-Rao Lower Bound for Proposed Method

In order to evaluate the estimation accuracy in the framework of sparse Bayesian inference, Ranjitha proposed the Bayesian Cramér-Rao lower bound (BCRB) which is the counterpart of Cramér-Rao lower bound [33]. In the following part, the BCRB of our proposed 3D reconstruction method is presented.

Denote the unknowns to be estimated as $\theta =[\sigma ;\delta_{s}]$, the BCRB of $\delta_{s}$ can be calculated by the following expression [33]:

(15) $$-E_{y,\theta }\left[\frac{\partial }{\partial \delta_{s}}{\left\{\frac{\partial \log p(y,\theta ;\eta ,\alpha )}{\partial \delta_{s}}\right\}}^{T}\right]$$

where:

(16) $$p(y,\theta ;\eta ,\alpha )=p(y|\sigma ,\delta_{s};\eta )p(\sigma ;\alpha )p(\delta_{s})$$

By substituting Equation (16) into (15), we can get the following expression after trivial manipulations:

(17) −Ey,θ[∂∂δs{∂logp(y,θ;η,α)∂δs}T]=2ηRe{Φ1HΦ⊙diag(α)}

More details about the BCRB can be found in [33]. According to Equation (17), BCRB of scatterer’s position estimation is:

(18) $$\mathrm{BCRB}(\delta_{s_{i}})=\frac{\eta }{2{\left(\Phi_{1}{}^{H}\Phi_{1}\right)}_{i,i}\alpha_{i}}=\frac{1}{2{\left(\Phi_{1}{}^{H}\Phi_{1}\right)}_{i,i}{\mathrm{SNR}}_{i}}$$

where we define ${\mathrm{SNR}}_{i}=\alpha_{i}/\eta$. From above equation, it is obvious that the estimation accuracy is inverse proportion to the signal to noise ratio (SNR). Besides, it is also influenced by the property of the matrix $\Phi_{1}$. Considering the expression of elements of $\Phi_{1}$, the BCRB can be further expressed as:

(19) $$\mathrm{BCRB}(\delta_{s_{i}})=\sqrt{\frac{\lambda^{2}R_{0}^{2}}{16\pi^{2}{\mathrm{SNR}}_{i}\sum_{i=1}^{P}{\left(\Delta b_{i}\right)}^{2}}}=\frac{\lambda R_{0}}{4\pi }\sqrt{\frac{1}{{\mathrm{SNR}}_{i}\sum_{i=1}^{P}{\left(\Delta b_{i}\right)}^{2}}},\forall i\in \{1,2\ldots ,L\}$$

where $\Delta b_{i}$ is the distance between the *i*th APC and center of all the APCs. As can be seen from Equation (19), the BCRB is proportion to the distance of target. In order to improve estimation accuracy, a properly designed APC distribution will be useful given fixed SNR and number of antennas.

## 4. Experimental Results

In this section, we use both simulated and measured data to analyze performance of our proposed algorithm. In Section 4.1, an experiment was conducted and the results of model order selection with measured data are shown. Besides, simulation results are presented to analyze performance of the proposed method. The super-resolution ability along with the normalized mean square error (NMSE) of off-grid mismatch estimation are presented in Section 4.2. In Section 4.3, the reconstructed 3D images of a Boeing-737 are presented to validate the practicality of the proposed algorithm.

### 4.1. Array ISAR System Configuration and Model Selection

**Figure 3:**
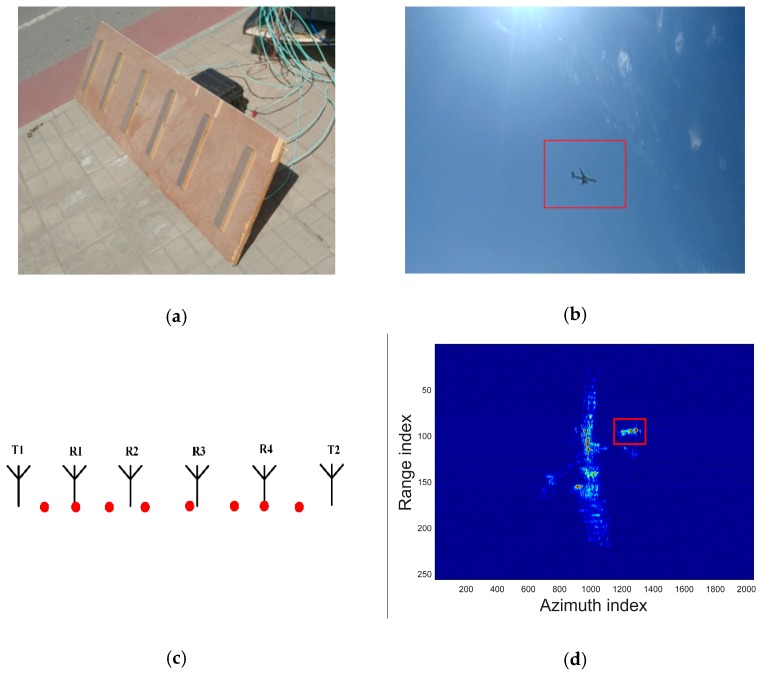
Array ISAR system configuration and ISAR images. (**a**) array ISAR system; (**b**) observed airplane; (**c**) distribution of APCs; (**d**) ISAR image by one of the channel.

To evaluate the feasibility of our proposed algorithm, we conducted an array ISAR imaging experiment near the Beijing International Airport. System parameters are shown in Table 1. Figure 3a,b present the array ISAR system and the observed airplane, respectively. The elements arrangement of this system are shown in Figure 3c. There are two transmitters and four receivers. The eight red solid dots stand for the APCs. With these APCs, we can use the multi-channel echo signal to obtain eight ISAR images. Figure 3d is one of these images and the area inside the red rectangle is where angular glint appears. Inside this area, the synthesis scatterers are consist of multiple dominant scatterers which are hard to distinguish by traditional interferometric ISAR technique.

**Figure 4:**
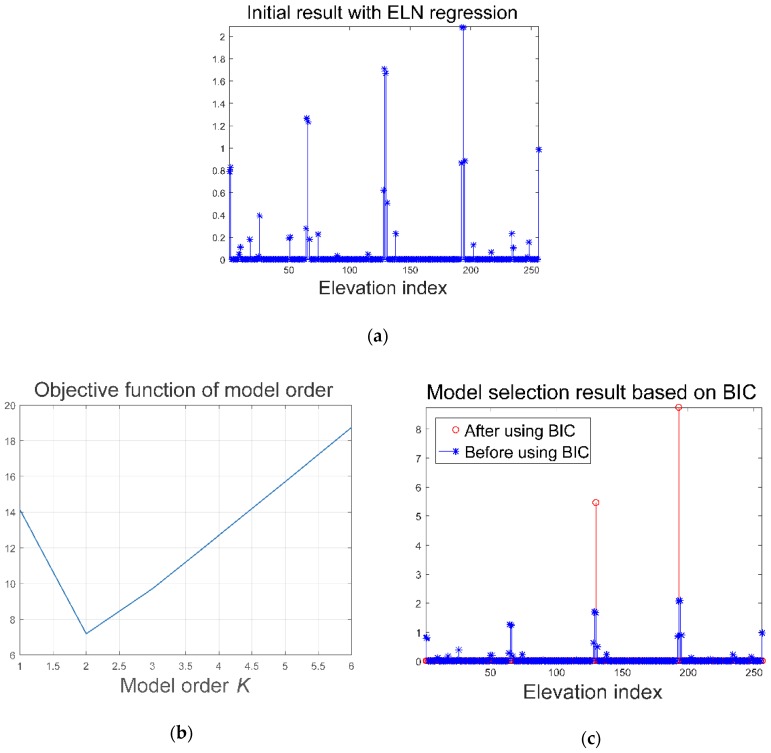
Model order selection results. (**a**) Initial result of the elastic net regression; (**b**) relations between model order *K* and the objective function; (**c**) optimized result after using BIC criterion.

With the eight channels echo signal, we first conduct the elastic net estimation to realize model order selection. Take the pixel (1291, 93) in the red rectangle in Figure 3d as an example, Equation (4) is utilized in which the weights $\lambda_{1},\lambda_{2}$ have been adjusted to get the best performance. The results are presented in Figure 4a. As can be seen, there are lots of outliers in the reconstructed result because of noise and the off-grid mismatch. Based on this result, model selection is completed using Equation (7). Since the number *K* of dominant scatterers is usually small, we use the function in (7) to evaluate the goodness of different *K*. The relations between model order *K* and the objective function are presented in Figure 4b. When K equals two, the objective function is minimal. With this estimation, the reconstructed reflectivity vector $\sigma_{\mathrm{ELN}}$ is got. A comparison between the reconstructed reflectivity distribution before and after model order selection is presented in Figure 4c. Although the result $\sigma_{\mathrm{ELN}}$ usually still suffers from the off-grid mismatch, it can be used as an initial input of the sparse Bayesian inference procedure.

### 4.2. Performance Analysis with Simulations

Since the observed plane is uncooperative and the distribution of dominant scatterers are not a known prior, it is difficult to evaluate performance of our proposed method. Toward this end, we use simulations instead and the simulation parameters are the same as Table 1.

**Figure 5:**
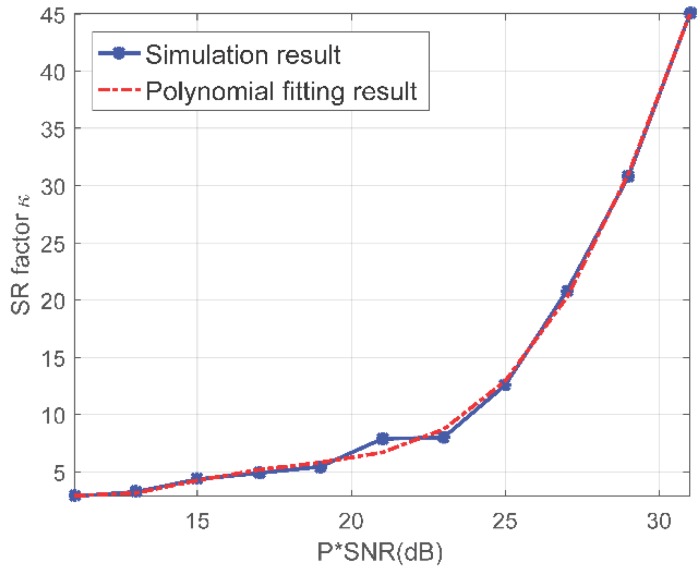
Super-resolution factor versus different P∗SNRs.

Inspired by the results in [34], we analyze the super-resolution ability of proposed method. The super-resolution (SR) factor is defined as $\kappa =\rho_{Rayleigh}/\rho$ in which $\rho_{Rayleigh}$ is the Rayleigh resolution decided by the baseline length and ρ is the resolution by proposed method. It is shown that κ is influenced by the product of SNR and number of APCs *P*. Figure 5 presents the SR factor with varying P*SNR and no amplitude or phase error. We refer readers to [27,34] for more explanations about the relations between κ and P*SNR. However, since the proposed method realize super-resolution imaging with multi-channel data, amplitude and phase error among different channels will have influence on the super-resolution imaging performance. With the parameters in Table 1, we further discuss the relations between SR factor and different amplitude and phase errors. Considering that the number of APCs is eight in our system and that the SNR is calculated to be 20.26 dB according to the ISAR images, we set P*SNR to 29 dB in the following simulations.

**Figure 6:**
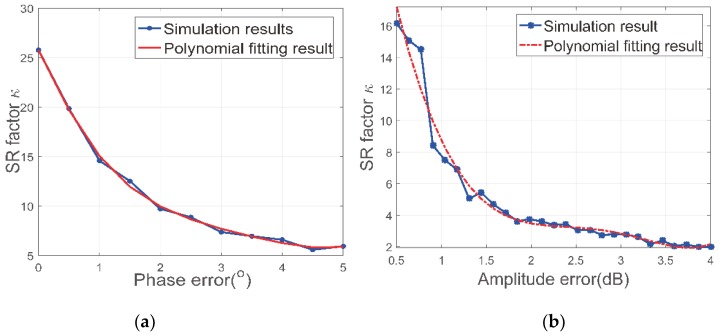
Super-resolution factor versus different kind of error (**a**) SR factor with respect to different phase error; (**b**) SR factor with respect to different amplitude error.

Assuming that the amplitude and phase error obey Gaussian distribution with different variances, we analyze the super-resolution power by Monte Carlo simulations. The number of Monte Carlo trails is set to 1000 and results are presented in Figure 6. In Figure 6a, it can be seen that the SR factor decreases with the phase error increases. From Figure 5, the SR factor is around 31 when there are none phase or amplitude error. The appearance of the phase error spoils the super-resolution ability to some extent. In our radar system, the phase error can be restricted to around 0.5 degrees, which means the SR factor will be reduced from 31 to 20 according to Figure 6a. With P*SNR being 29 dB and phase error being 0.5 degrees, we further investigate the relations between SR factor and amplitude error in Figure 6b. As expected, the amplitude error will also introduce reduction of the SR factor. When the amplitude is around 0.5 dB which is typical in our radar system, the SR factor will finally reduce to around 15. We adopt polynomial fitting in this section and the coefficients are summarized in Table 2. Although these coefficients are obtained by simulations, they can serve as a reference for further analysis about the super-resolution ability of the array ISAR system. The results in Figure 5 and Figure 6 suggests that the SR ability of proposed method will be influenced by both phase and amplitude error. However, the SBI based imaging method can still achieve a satisfactory SR ability with typical value of those errors.

**Figure 7:**
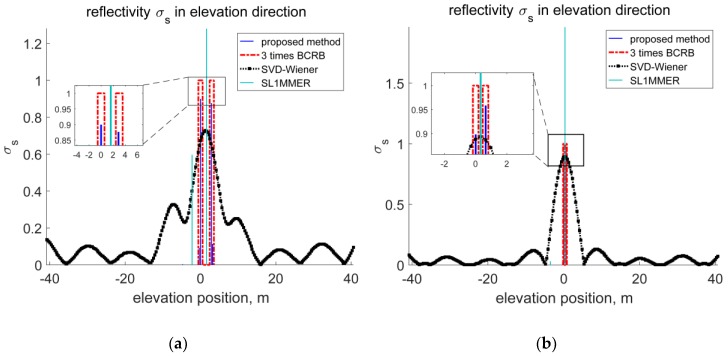
Comparison of reconstructed reflectivity profiles in elevation direction between proposed method and SVD-Wiener. (**a**) Scatterers’ distance 3 m, SNR = 10 dB, BCRB = 0.1875 m; (**b**) Scatterers’ distance 0.63 m, SNR = 20 dB, BCRB = 0.0593 m.

Based on the APC distribution, the maximal baseline is 1.31 m. The Rayleigh resolution in the elevation direction is calculated to be 6.3825 m. According to Equation (19), the BCRB of scatterer’s position estimation is 0.1875 m and 0.0593 m when SNR equals 10 dB and 20 dB, respectively. Assuming that there are two dominant scatterers in the same range-Doppler unit and their reflectivity coefficient are both unit, Figure 7 shows the reconstructed results with different SNR and scatterers’ distance. Traditional imaging algorithms in the elevation direction can mainly be divided into three categories, i.e., the Fourier transform based algorithms [35], spectral analysis based algorithms [34,36] and compressed sensing based algorithms [27,37,38]. However, the performance of Fourier transform based algorithms is severely degraded by the unequally spaced baselines and we ignore the approach of Fourier transform here. In order to compare the resolution ability between proposed method and traditional methods, we choose a representative algorithm from the latter two categories. The SVD-Wiener algorithm [34] and the SL1MMER algorithm [38], which stands for Scale-down by L1 norm Minimization, Model selection, and Estimation Reconstruction, are chosen as references. In Figure 7a, the two scatterers are located at 0 m and 3 m and the SNR is set to be 10 dB. In Figure 7b, the distance is 0.63 m and SNR equals 20 dB. As can be seen from these figures, compared with the SVD-Wiener algorithm, scatterers within a Rayleigh limit can be successfully distinguished by proposed method and it can realize ten times SR imaging in elevation direction. As for the SL1MMER algorithm, the mismatch problem described in Section 3 has a severe influence on the reconstruction performance. The multiplicative noise introduced by the mismatch makes it hard to get an exact estimation of scatterers’ position and RCS. The proposed algorithm solves this by using the sparse Bayesian inference technique and a better result is obtained. Besides, the estimations of scatterers’ position are within ±3 times BCRB around the true position, which proves the accuracy.

After above procedures, dominant scatterers in a single pixel can be distinguished from a synthesis scatterer and the 3D point cloud can be extracted. Since the 3D point cloud is reconstructed from multi-channel ISAR images, which means that the underdetermined problem in Equation (3) needs to be solved for every single pixel of the 2D images. This will make the post processing very time consuming. Considering that the amplitude of pixel where synthesis scatterer emerges is larger than where there is no synthesis scattterer, a threshold is set to find pixels in the ISAR images where angular glint effect exists. Then the sparse Bayesian inference technique is only applied to these pixels and the overall computation time can be effectively reduced. With the measured data in the manuscript, the overall computation time by proposed method is around five times as much as the interferometric approach. Although our proposed method will need more computation time, it can effectively solve the problem of synthesis scatterers and the dominant scatterers can be successfully distinguished. It is mentioned in the introduction that three-dimensional imaging method based on sequence ISAR images needs a long observation time because a set of different view angles provide information for estimation of the 3D position of each target scattering center [16]. Thus a few coherent processing interval (CPI) are needed for a sequence of ISAR images [19]. As a comparison, the array ISAR three-dimensional imaging method needs only one CPI because the data acquisition is fulfilled by all the channels at the same time. And the imaging duration can be reduced by proposed method compared with the sequence ISAR approach.

**Figure 8:**
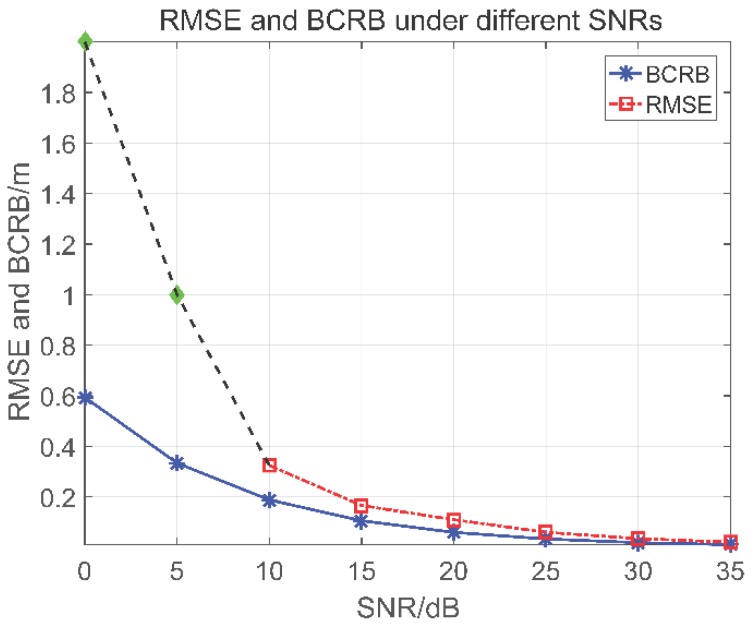
Comparison between RMSE and BCRB under different SNRs.

In order to further demonstrate the effectiveness of the proposed method, the root mean square error (RMSE) of estimated scatterer’s position and its corresponding BCRB are compared. The RMSE is defined as follows:

(20) $$\mathrm{RMSE}(s)=\sqrt{1/T\sum_{t=1}^{T}{\left({\hat{s}}_{t}-s_{t}\right)}^{2}}$$

in which *T* is the number of Monte-Carlo trails. In this simulation, only a single scatterer which is located at 6.6 m is considered and *T* is set to 500. Figure 8 presents the RMSE and BCRB under different SNRs. In this figure, the RMSE of estimated position approximates the BCRB when the SNR increases. However, when the SNR is less than 10 dB, the sparse recovery fails and the scatterer’s position cannot be properly estimated.

### 4.3. 3D Reconstruction Results Based on Measured Data

**Figure 9:**
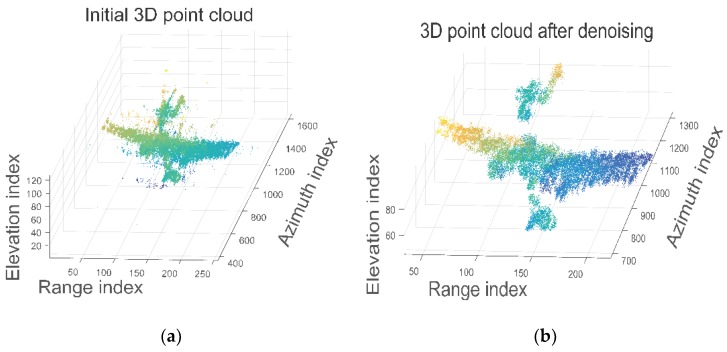
3D point cloud by proposed method. (**a**) Result without point cloud filtering; (**b**) Result after point cloud filtering based on k-nearest neighbor.

By applying the proposed method to real radar data, we can get reconstructed 3D image of the target. With the eight channels echo signal we can get eight ISAR images. With the result obtained after model selection in Section 3.1, the sparse Bayesian inference technique is applied to fulfill super-resolution 3D imaging. Since there are some hyper-parameters used in the process, we set ρ=0.01, $c=d=10^{-5}$ and initialize $\sigma^{\left(0\right)}=\sigma_{\mathrm{ELN}}$ and $\eta^{\left(0\right)}={\left\|y-\Phi \sigma \right\|}^{2}/P$. The value of ρ, *c* and *d* are set close to zero in order to get a broader prior [39]. The reconstructed 3D point cloud is presented in Figure 9.

In Figure 9a, it can be seen that there are lots of outliers outside the boundary of the target due to the noise. In order to get a better 3D model, we adopt point cloud filtering based on *k*-nearest neighbor algorithm. Denote a scatterer in Figure 9a as *I* and a set containing its *k*-nearest scatterers as Ω, we can calculate the mean distance between the scatterer *I* and the scatterers in Ω:

(21) $$d_{I}(K)=\frac{1}{K}\sum_{k}{\left\|I-J\right\|}_{2}(J\in \Omega )$$

The average distance is then compared with a predefined threshold ξ. If $d_{I}\left(K\right)$ is larger than ξ, then scatterer *I* is regarded as noise and will be discarded from the point cloud. After this procedure, the optimized 3D point cloud is presented in Figure 9b. It is obvious that the outliers are removed.

**Figure 10:**
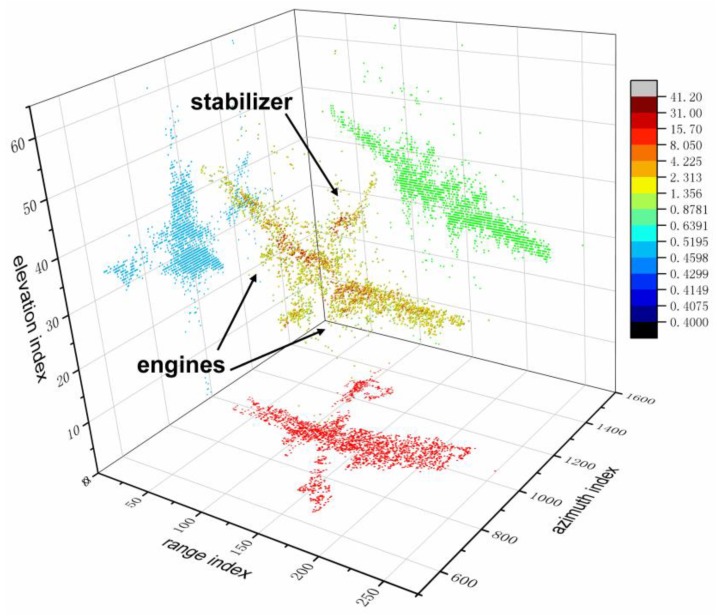
3D reconstruction results of airplane with Ku band array ISAR system.

The final 3D result is shown in Figure 10 in which the color of scatterers stands for the RCS. In the center of this figure is the reconstructed 3D model while the three views of this model are presented with different colors. Among these projections, the red one is the projection of the target onto the range-Doppler plane. Along with other projections presented, it can be seen that multiple scatterers in the same range-Doppler unit can be well distinguished by proposed algorithm. Besides, features of the airplane such as engines and stabilizers can be extracted as is shown in Figure 10. These results verify that the proposed method outperforms traditional spectral analysis methods and can effectively solve the problem of synthesis scatterers in real scene. The proposed method provides a solution to the problem of synthesis scatterers and can extract the three-dimensional model of target of interest. Compared with 2D radar images, 3D models can provide the structure of aircraft and the pose is also obtained. With these information, this technique can be applied in target recognition and fault detection. However, there also exist some shortcomings. The proposed three-dimensional imaging method is based on multi-channel ISAR images, which means that the quality of the ISAR images is of fundamental importance to the 3D reconstruction. Its 3D reconstruction performance may be spoiled when the targets are high maneuvering since target’s mobility during the coherent processing interval (CPI) will have influence on the quality of the ISAR images, which will finally affect the performance of proposed three-dimensional imaging method. Overall, this study solves the problem of synthesis scatterers and makes some contributions to 3D imaging of air targets.

## 5. Conclusions

The problem of synthesis scatterers emerges when multiple dominant scatterers are with the same range and Doppler frequency. Traditional interferometric technique based three-dimensional imaging method cannot resolve multiple scatterers from a synthesis scatterer. The problem with this phenomenon is that the 3D structure of a target cannot be successfully captured, which will make it difficult for target recognition. In order to solve this problem, a 3D imaging method based on sparse Bayesian inference with an array ISAR system is proposed. Considering the characteristics of the over-complete dictionary and the sparseness of imaging scene, the elastic net estimation along with sparse Bayesian inference are chosen to realize super-resolution 3D imaging. In the first step, the elastic net regression is first used to obtain an initial estimation, then model selection with BIC criterion is adopted to remove the outliers and get an accurate estimation of the number *K* of dominant scatterers. With this result, the sparse Bayesian inference is utilized to get the final estimation of reflectivity function free of multiplicative noise caused by grid mismatch. The proposed method is applied to both simulated and measured data to validate its practicality. The results obtained show that the proposed method can realized ten times super-resolution imaging in the elevation direction. As a comparison, the SVD-Wiener method cannot distinguish scatterers inside a Rayleigh limit and the performance of SL1MMER algorithm is degraded by the multiplicative noise caused by basis mismatch. Besides, quantitative analyses are provided to illustrate the relations between SR factor and phase and amplitude errors. To verify the effectiveness of proposed method, the RMSE is compared with its corresponding BCRB. Taken together, these results suggest that the proposed method can effectively solve the problem of synthesis scatterer and provide a new understanding of 3D imaging of aircrafts.

## Figures and Tables

**Table 1:** Array ISAR system configuration.

Parameter	Symbol	Value
Carrier frequency	*f_c_*	15 GHz
Bandwidth	*B*_w_	500 MHz
Pulse repetition frequency	*PRF*	1 KHz
Velocity of plane	*v*	63.5 m/s
Reference range	*R_ref_*	836.4 m
Number of APCs	*P*	8
Maximum baseline	$\Delta b_{max}$	1.31 m

**Table 2:** Polynomial approximation of the SR factors as a function of P*SNR, phase error and amplitude error.

	Zero-Order	1st Order	2nd Order	3rd Order	4th Order	5th Order
P*SNR	−10.091	1.0498	0.099758	−0.011345	2.8687 × 10^−4^	0
Phase error	25.083	−14.242	3.797	−0.17337	−0.097998	0.012569
Amplitude error	31.017	−35.754	13.942	−0.55709	−0.71587	0.10677
